# Non-contiguous finished genome sequence and description of *Clostridium dakarense* sp. nov.

**DOI:** 10.4056/sigs.4097825

**Published:** 2013-09-30

**Authors:** Cheikh Ibrahima Lo, Ajay Kumar Mishra, Roshan Padhmanabhan, Bissoume Samb, Amy Gassama Sow, Catherine Robert, Carine Couderc, Ngor Faye, Didier Raoult, Pierre-Edouard Fournier, Florence Fenollar

**Affiliations:** 1 Aix-Marseille Université, Unité de Recherche sur les Maladies Infectieuses et Tropicales Emergentes (URMITE), Marseille, France; 2 Unité de Bactériologie Expérimentale Institut Pasteur de Dakar 36, Dakar, Senegal; 3 Université Cheikh Anta Diop de Dakar, Laboratoire de Parasitologie générale, Fann (Senegal)

**Keywords:** *Clostridium dakarense*, genome, culturomics, taxono-genomics

## Abstract

*Clostridium dakarense* strain FF1^T^, is the type strain of *Clostridium dakarense* sp. nov., a new species within the genus *Clostridium*. This strain, whose genome is described here, was isolated from the fecal flora of a 4-month-old Senegalese child suffering from gastroenteritis. *C. dakarense* sp. nov. strain FF1^T^ is an obligate anaerobic Gram-positive bacillus. Here we describe the features of this organism, together with the complete genome sequence and annotation. The 3,735,762 bp long genome (1 chromosome but no plasmid) exhibits a G+C content of 27.98% and contains 3,843 protein-coding and 73 RNA genes, including 8 rRNA genes.

## Introduction

*Clostridium dakarense* strain FF1^T^ (= CSUR P243 = DSM 27086), is the type strain of *Clostridium dakarense* sp. nov. This bacterium is a Gram-positive, anaerobic, spore-forming, indole negative bacillus that was isolated from the stool of a 4-month-old Senegalese child suffering from gastroenteritis as part of a “culturomics” study aiming at cultivating individually all species within human feces.

The elevated cost and lack of intra- and inter-laboratory reproducibility of the “gold standard” of taxonomic tools. i.e. DNA-DNA hybridization and G+C content determination [1], put bacterial taxonomic classification in a precarious state. In addition, the internationally-validated cutoff values of 16S rRNA sequence comparison [2] do not apply to all validly published genera and species. Recently, high throughput genome sequencing and mass spectrometric analyses of bacteria have allowed unprecedented access to a wealth of genetic and proteomic information [3]. As a consequence, we proposed to use a polyphasic approach [4] to describe new bacterial taxa, including genome sequence, MALDI-TOF spectrum and main phenotypic characteristics [5-11].

The genus *Clostridium* (Prazmowski, 1880), classified among the *Firmicutes*, was created in 1880 [12] and consists of obligate anaerobic rod-shaped bacilli capable of producing endospores [12]. More than 180 *Clostridium* species have been described to date [13]. Members of the genus *Clostridium* are mostly environmental bacteria or associated with the commensal digestive flora of mammals, but several are major human pathogens, including *C. botulinum*, *C. difficile*, *C. tetani* and *C. perfringens* [14,15]. A few species, such as *C. butyricum* and *C. pasteurianum*, fix nitrogen and have gained importance in agricultural and industrial applications [16,17].

Here we present a summary classification and a set of features for *C. dakarense* sp. nov. strain FF1^T^ (= CSUR P243 = DSM 27086) together with the description of the complete genomic sequencing and annotation. These characteristics support the circumscription of the species *C. dakarense* sp. nov.

## Classification and features

**Figure 1:**
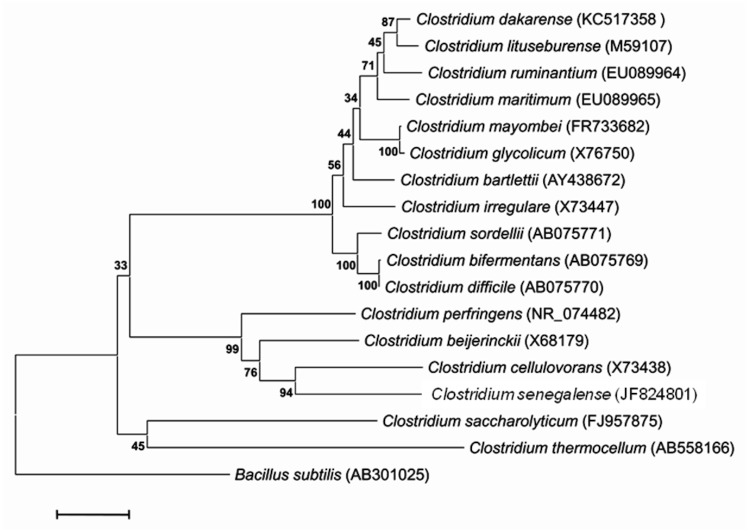
Phylogenetic tree highlighting the position of *C. dakarense* sp. nov. strain FF1^T^ relative to other type strains within the *Clostridium* genus. GenBank accession numbers are indicated in parentheses. Sequences were aligned using CLUSTALW, and phylogenetic inferences obtained using the maximum-likelihood method within the MEGA software. Numbers at the nodes are bootstrap values obtained by repeating 500 times the analysis to generate a majority consensus tree. *Bacillus subtilis* was used as an outgroup. The scale bar represents a 2% nucleotide sequence divergence.

A stool specimen was collected from a 4-month-old Senegalese child suffering from gastroenteritis. Informed consent was obtained from the child’s parents and approval from the ethics committee from the Institut Federatif de Recherche 48 (Faculté de Médecine, Marseille, France). The fecal specimen was preserved at -20°C after collection and sent to Marseille. Strain FF1^T^ (Table 1) was isolated in July 2011 by anaerobic cultivation on 5% sheep blood-enriched Columbia agar (BioMerieux, Marcy l’Etoile, France). This strain exhibited a 96.90% 16S rRNA nucleotide sequence similarity with *C. lituseburense*, the phylogenetically closest validated *Clostridium* species (Figure 1). Although sequence similarity of the 16S rRNA is not uniform across taxa, this value was lower than the 98.7% threshold recommended by Stackebrandt and Ebers to delineate a new species without carrying out DNA-DNA hybridization [30]. In addition, it was consistent with 16S rRNA identity values observed among validated species within the *Clostridium* genus that range from 78.4 to 99.8%.

**Table 1:** Classification and general features of *Clostridium dakarense* strain FF1^T^ according to the MIGS recommendations [18].

**MIGS ID**	**Property**	**Term**	**Evidence code^a^**
		Domain *Bacteria*	TAS [19]
		Phylum *Firmicutes*	TAS [20-22]
		Class *Clostridia*	TAS [23,24]
	Current classification	Order *Clostridiales*	TAS [25,26]
		Family *Clostridiaceae*	TAS [25,27]
		Genus *Clostridium*	TAS [12,25,28]
		Species *Clostridium dakarense*	IDA
		Type strain FF1	IDA
	Gram stain	Positive	IDA
	Cell shape	Rod-shaped	IDA
	Motility	Motile	IDA
	Sporulation	Sporulating	IDA
	Temperature range	Mesophile	IDA
	Optimum temperature	37°C	IDA
MIGS-6.3	Salinity	Growth in BHI medium + 5% NaCl	IDA
MIGS-22	Oxygen requirement	Anaerobic	IDA
	Carbon source	Unknown	NAS
	Energy source	Unknown	NAS
MIGS-6	Habitat	Human gut	IDA
MIGS-15	Biotic relationship	Free living	IDA
MIGS-14	Pathogenicity Biosafety level Isolation	Unknown 2 Human feces	NAS
MIGS-4	Geographic location	Senegal	IDA
MIGS-5	Sample collection time	June 2011	IDA
MIGS-4.1	Latitude	13.7167	IDA
MIGS-4.1	Longitude	- 16.4167	IDA
MIGS-4.3	Depth	Surface	IDA
MIGS-4.4	Altitude	51 m above sea level	IDA

**^a^**Evidence codes - IDA: Inferred from Direct Assay; TAS: Traceable Author Statement (i.e., a direct report exists in the literature); NAS: Non-traceable Author Statement (i.e., not directly observed for the living, isolated sample, but based on a generally accepted property for the species, or anecdotal evidence). These evidence codes are from the Gene Ontology project [29]. If the evidence is IDA, then the property was directly observed for a live isolate by one of the authors or an expert mentioned in the acknowledgements.

**Figure 2:**
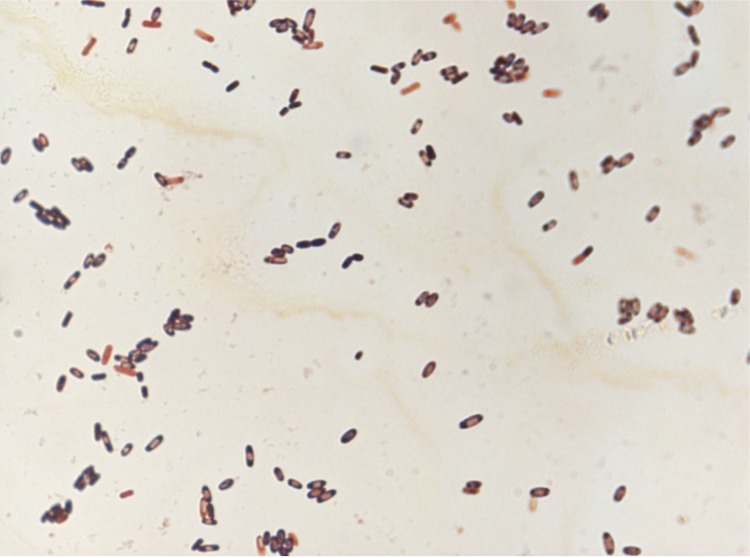
Gram staining of *C. dakarense* sp. nov. strain FF1^T^.

**Figure 3:**
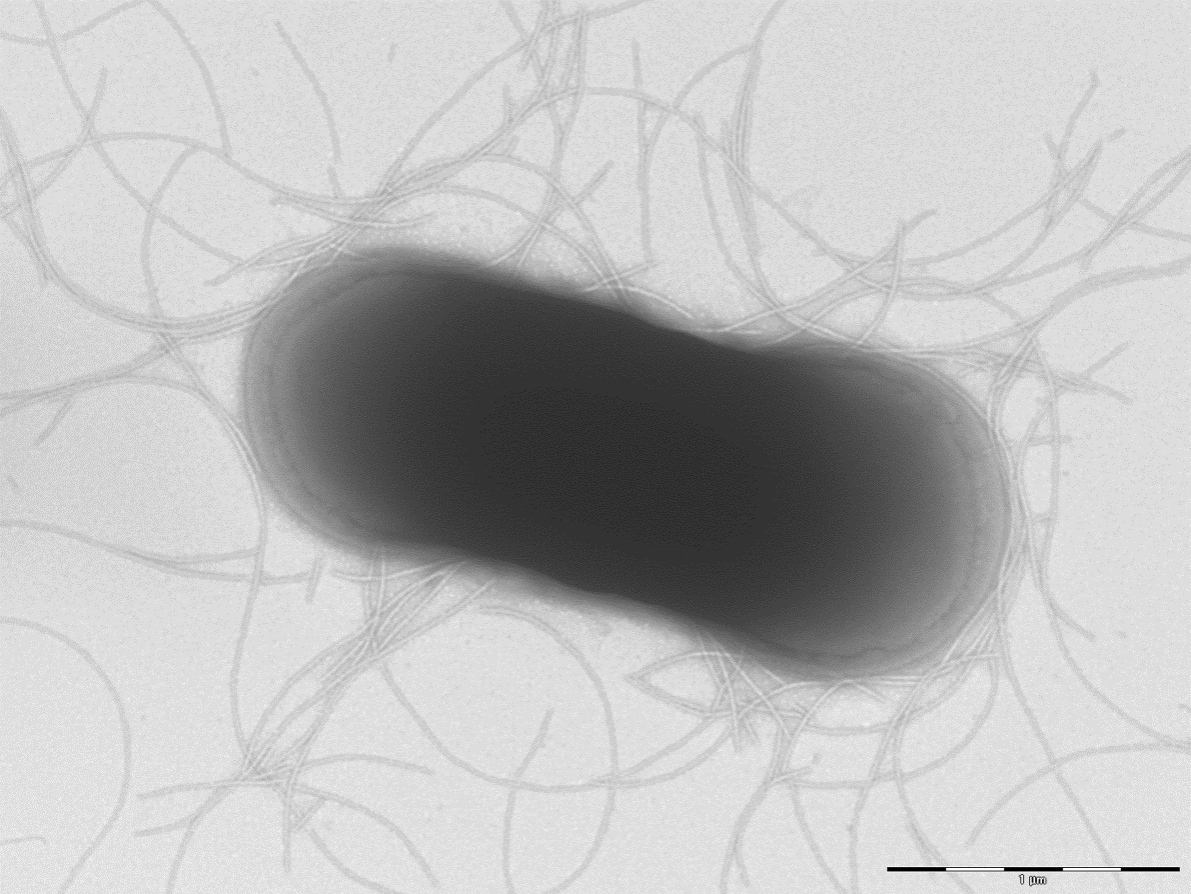
Transmission electron microscopy of *C. dakarense* sp. nov. strain FF1^T^, using a Morgani 268D (Philips) at an operating voltage of 60kV. The scale bar represents 1 µm.

Different growth temperatures (25, 30, 37, 45 and 56°C) were tested. Growth was observed between 25 and 37°C, with optimal growth at 37°C after 24 hours of inoculation in anaerobic conditions. Colonies were 1.5 mm in diameter and opaque and smooth appearance on blood-enriched Columbia agar. Growth of the strain was tested under anaerobic and microaerophilic conditions using GENbag anaer and GENbag microaer systems, respectively (BioMerieux), and under aerobic conditions, with or without 5% CO_2_. The strain growth was obtained only in anaerobic conditions. Gram staining showed rod-shaped Gram-positive bacilli able to form spores (Figure 2). The motility test was positive. Cells grown on agar have a mean diameter of 1.2 µm (Figure 3).

Strain FF1^T^ exhibited neither catalase nor oxidase activities. Using API Rapid ID 32A (BioMerieux, Marcy l’Etoile), a positive reaction were observed for arginine dihydrolase, N-acetyl-β-glucosaminidase and pyroglutamic acid arylamidase. Negative reactions were observed for urease, indole and nitrate reduction. Using API 50 CH (BioMerieux, Marcy l’Etoile), positive reactions were observed for galactose, glucose, maltose and saccharose fermentation and negative reaction were observed for ribose, lactose and fructose. *C. dakarense* is susceptible to amoxicillin, metronidazole, vancomycin, imipenem and rifampicin and resistant to trimethoprim/ sulfamethoxazole. When compared with representative species from the genus *Clostridium*, *C. dakarense* strain FF1^T^ exhibited the phenotypic differences detailed in Table 2.

**Table 2:** Differential characteristics of *C. dakarense* sp. nov. strain FF1^T^ (Cda)

**Properties**	**CDa**	**CBa**	**CBe**	**CC**	**CDi**	**CG**	**CP**	**CSa**	**CSe**	**CT**
Cell diameter (µm)	1.2	1.5	1.7	2.5	3.0	0.4-1.0	1.3	3.0	1.1	2.5
Oxygen requirement	-	-	-	-	-	-	-	-	-	na
Pigment production	-	-	-	-	+	+	+	na	-	+
Gram stain	+	+	V	-	+		+	-	+	-
Salt requirement	-	na	na	na	na	-	-	na	-	na
Motility	+	-	+	-	+	+	-	-	+	-
Endospore formation	+	+	+	+	+	+	w	+	+	+
										
**Production of**										
Acid phosphatase	+	+	na	na	na	na	+	na	na	na
Catalase	-	-	-	-	na	na	na	na	-	na
Oxidase	-	na	na	na	na	na	na	na	-	na
Nitrate reductase	-	-	-	na	-	-	+	+	-	-
Urease	-	-	-	na	na	na	na	na	-	na
β-galactosidase	-	+	na	na	na	-	+	na	-	na
										
**Acid from**										
L-Arabinose	-	na	+	-	-	-	-	+	na	na
Ribose	-	+	-		-	-	+	w	na	na
Mannose	-	-	+		+	-	+	na	na	na
Mannitol	-	+	+	+	+	-	-	w	na	na
Sucrose	-	+	+	+	+	-	+	w	na	na
D-glucose	+	+	+	+	na	+	+		na	na
D-fructose	-	+	+	+	+	+	+	+	na	na
D-maltose	+	+	+	+	-	+	+	w	na	na
D-lactose	-	na	+	+	-	-	+	w	na	na
										
**Hydrolysis of**										
Gelatin	na	-	+	-	na	-	na	na	na	+
Starch	na	na	+	-	-	-	+	-	na	
										
**G+C content (mol%)**	27.98	29.8	28	27	28	29	27	28	26.8	39
										
**Habitat**	Human gut	Human gut	Human gut	Poplar wood	Human gut	Mud, wastewater	Colonic flora	Sewage sludge	Human gut	Sewage sludge

*C. bartlettii* (CBa), *C. beijerinckii* (CBe), *C. cellulovorans* (CC), *C. difficile* (CDi), *C. glycolicum* (CG), *C. perfringens* (CP), *C. saccharolyticum* (CSa), *C. senegalense* (CSe) and *C. thermocellum* (CT).

na = data not available; w = weak, v = variable reaction

**Figure 4:**
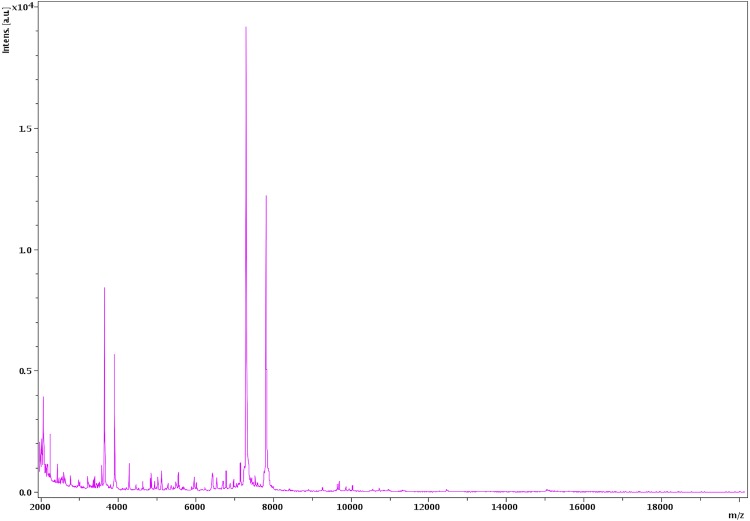
Reference mass spectrum from *C. dakarense* strain FF1^T^. Spectra from 18 individual colonies were compared and a reference spectrum was generated.

**Figure 5:**
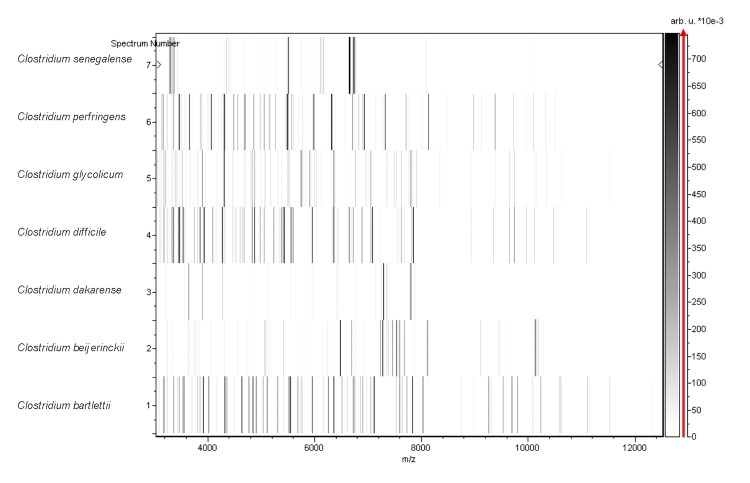
Gel view comparing *C. dakarense* sp. nov. strain FF1^T^ spectra with other members of the *Clostridium* genus (*C. bartlettii*, *C. beijerinckii*, *C. difficile*, *C. glycolicum*, *C. perfringens*, *C. senegalense*). The Gel View displays the raw spectra of all loaded spectrum files arranged in a pseudo-gel like look. The x-axis records the m/z value. The left y-axis displays the running spectrum number originating from subsequent spectra loading. The peak intensity is expressed by a Gray scale scheme code. The color bar and the right y-axis indicate the relation between the color a peak is displayed with and the peak intensity in arbitrary units.

Matrix-assisted laser-desorption/ionization time-of-flight (MALDI-TOF) MS protein analysis was carried out as previously described [31]. Briefly, a pipette tip was used to pick one isolated bacterial colony from a culture agar plate, and to spread it as a thin film on a MTP 384 MALDI-TOF target plate (Bruker Daltonics, Leipzig, Germany). Eighteen distinct deposits were made for strain FF1^T^ from eighteen isolated colonies. Each smear was overlaid with 2 µL of matrix solution (saturated solution of alpha-cyano-4-hydroxycinnamic acid) in 50% acetonitrile, 2.5% tri-fluoracetic-acid, and allowed to dry for five minutes. Measurements were performed with a Microflex spectrometer (Bruker). Spectra were recorded in the positive linear mode for the mass range of 2,000 to 20,000 Da (parameter settings: ion source 1 (IS1), 20 kV; IS2, 18.5 kV; lens, 7 kV). A spectrum was obtained after 675 shots at a variable laser power. The time of acquisition was between 30 seconds and 1 minute per spot. The eighteen spectra were imported into the MALDI BioTyper software (version 2.0, Bruker) and analyzed by standard pattern matching (with default parameter settings) against the main spectra of 4,706 bacteria including 216 spectra from validly published species of *Clostridium*, that are part of the reference data contained in the BioTyper database. The method of identification included the m/z from 2,000 to 20,000 Da. For every spectrum, 100 peaks at most were taken into account and compared with spectra in the database. A score enabled the identification, or not, from the tested species: a score > 2 with a validly published species enabled the identification at the species level, and a score < 1.7 did not enable any identification at the genus level. For strain FF1^T^, the maximal obtained score was lower than 1.9, thus suggesting that our isolate was not a member of a known species. We added the spectrum from strain FF1^T^ to our database for future reference (Figure 4). Finally, the gel view allows us to highlight the spectrum differences with other members of the genus *Clostridium* (Figure 5).

## Genome sequencing information

### Genome project history

The organism was selected for sequencing on the basis of its phylogenetic position and 16S rRNA similarity to other members of the genus *Clostridium*, and is part of a “culturomics” study of the human digestive flora aiming at isolating all bacterial species within human feces. It was the 94^th^ genome of a *Clostridium* species and the first genome of *Clostridium dakarense* sp. nov. The Genbank accession number is CBTZ00000000 and consists of 257 contigs. Table 3 shows the project information and its association with MIGS version 2.0 compliance [32].

**Table 3:** Project information

**MIGS ID**	**Property**	**Term**
MIGS-31	Finishing quality	High-quality draft
MIGS-28	Libraries used	One 454 paired end 3-kb library
MIGS-29	Sequencing platforms	454 GS FLX Titanium
MIGS-31.2	Fold coverage	35
MIGS-30	Assemblers	Newbler version 2.5.3
MIGS-32	Gene calling method	Prodigal
	Genbank ID	CBTZ00000000
	Genbank Date of Release	
MIGS-13	Project relevance	Study of the human gut microbiome

### Growth conditions and DNA isolation

*C. dakarense* sp. nov. strain FF1^T^ (= CSUR P243 = DSM 27086), was grown anaerobically on sheep blood-enriched Columbia agar medium at 37°C. Eight petri dishes were spread and resuspended in 4x100µl of G2 buffer (EZ1 DNA Tissue kit, Qiagen). A first mechanical lysis was performed by glass powder on the Fastprep-24 device (Sample Preparation system) from MP Biomedicals, USA) using 2x20 seconds cycles. DNA was then treated with 2.5 µg/µL lysozyme (30 minutes at 37°C) and extracted through the BioRobot EZ 1 Advanced XL (Qiagen). The DNA was then concentrated and purified on a Qiamp kit (Qiagen). DNA concentration was 70.7ng/µl as determined by the Genios Tecan fluorometer, using the Quant-it Picogreen kit (Invitrogen).

### Genome sequencing and assembly

This project was loaded twice on a 1/4 region for the paired-end application and once on a 1/8 region for the shotgun on PTP Picotiterplates. The shotgun library was constructed with 500 ng of DNA as described by the manufacturer (Roche) with the GS Rapid library Prep kit. For the paired-end sequencing, 5 µg of DNA was mechanically fragmented on the Hydroshear device (Digilab, Holliston, MA, USA) with an enrichment size of 3-4kb. The DNA fragmentation was visualized using an Agilent 2100 BioAnalyzer on a DNA labchip 7500, which yield an optimal size of 3.6 kb. The library was constructed according to the 454_Titanium paired-end protocol and manufacturer. Circularization and nebulization were performed and generated a pattern with an optimum at 561 bp. After PCR amplification through 15 cycles followed by double size selection, the single stranded paired end library was then quantified with Quant-it Ribogreen kit (Invitrogen) on the Genios_Tecan fluorometer at 52 pg/µL. The library concentration equivalence was calculated as 1.7E+08 molecules/µL. The library was stored at -20°C until use.

The shotgun library was clonally amplified with 3cpb in 3 emPCR reactions and the paired end library was amplified with lower cpb (1cpb) in 4 emPCR reactions with the GS Titanium SV emPCR Kit (Lib-L) v2. The yield of the emPCR was 5.37% for the shotgun reads and 19.27% for the paired-end reads, according to the quality expected by the range of 5 to 20% from the Roche procedure. A total of 340,000 beads from the 1/8 region of the shotgun reads and 790,000 beads from the 1/4 region of the paired-end reads were loaded on the GS Titanium PicoTiterPlates (PTP Kit 70×75) and sequenced with the GS Titanium Sequencing Kit XLR70.

The runs were performed overnight and then analyzed on the cluster through the gsRunBrowser and gsAssembler_Roche. The global 383,079 passed filter sequences generated 96.50 Mb with a length average of 277 bp. These sequences were assembled using the Newbler software from Roche with 90% identity and 40 bp as overlap. Fourteen scaffolds and 257 large contigs (>1500bp) were obtained, for a genome size of 3,735,762 bp.

### Genome annotation

Open Reading Frames (ORFs) were predicted using Prodigal [33] with default parameters but the predicted ORFs were excluded if they spanned a sequencing gap region. The predicted bacterial protein sequences were searched against the GenBank database [34] and the Clusters of Orthologous Groups (COG) databases using BLASTP. The tRNAScanSE tool [35] was used to find tRNA genes, whereas ribosomal RNAs were found by using RNAmmer [36] and BLASTn against the GenBank database. Lipoprotein signal peptides and numbers of transmembrane helices were predicted using SignalP [37] and TMHMM [38] respectively. ORFans were identified if their BLASTP *E*-value was lower than 1e^-03^ for alignment length greater than 80 amino acids. If alignment lengths were smaller than 80 amino acids, we used an *E*-value of 1e-05. Such parameter thresholds have already been used in previous works to define ORFans. Artemis [39] was used for data management and DNA Plotter [40] was used for visualization of genomic features. Mauve alignment tool was used for multiple genomic sequence alignment and visualization [41].

To estimate the mean level of nucleotide sequence similarity at the genome level between *C. dakarense* and nine other members of the genus *Clostridium* (Table 6), we use the Average Genomic Identity of gene Sequences (AGIOS) home-made software. Briefly, this software combines the Proteinortho software [42] for detecting orthologous proteins between genomes compared two by two, then retrieves the corresponding genes and determines the mean percentage of nucleotide sequence identity among orthologous ORFs using the Needleman-Wunsch global alignment algorithm. *Clostridium dakarense* strain FF1^T^, was compared to *C. bartlettii* strain DSM 16795 (GenBank accession number NZ_DS499569), *C. beijerinckii* strain NCIMB 8052 (NC_009617), *C. cellulovorans* strain 743B (NC_014393), *C. difficile* strain 630 (NC8009089), *C. glycolicum* strain ATCC 14880 (ARES01000000), *C. perfringens* strain ATCC 13124 (BA000016), *C. saccharolyticum* strain WM1 (NC_014376), *C. senegalense* strain JC122^T^ (CAEV00000000), and *C. thermocellum* strain ATCC 27405 (CP000568).

**Table 6:** Numbers of orthologous proteins shared between genomes (upper right)

	CDa	CC	CBe	CP	CSe	CSa	CT	CBa	CG	CDi
CDa	**3,808**	1,045	1,230	1,089	1,131	1,013	806	1,324	1,690	1,203
CC	68.22	**4,254**	1,490	1,163	1,181	1,057	967	871	1,038	1,021
CBe	68.84	70.36	**5,020**	1,300	1,289	1,207	968	989	1,204	1,129
CP	70.02	70.43	72.15	**2,660**	1,168	920	777	845	1,005	1,147
CSe	69.91	70.37	70.82	70.13	**3,704**	930	821	856	1,134	1,008
CSa	61.94	62.50	62.44	62.22	62.05	**4,154**	854	833	1,004	998
CT	64.49	64.84	64.56	64.78	64.53	63.83	**3,173**	713	840	952
CBa	74.98	68.22	68.84	69.46	69.52	62.15	64.73	**2,787**	1,517	1,303
CG	75.70	68.28	68.83	69.49	69.57	62.26	64.59	76.04	**3,840**	1,568
CDi	71.34	69.57	68.52	71.52	65.49	66.37	64.32	74.45	74.50	**3,798**

average percentage similarity of nucleotides corresponding to orthologous protein shared between genomes (lower left) and numbers of proteins per genome (bold). CDa: *C. dakarense*; CC: *C. cellulovorans*; CBe: *C. beijerinckii*; CP: *C. perfringens*; CSe: *C. senegalense*; CSa: *C. saccharolyticum*; CT: *C. thermocellum*; CBa: *C. bartlettii*; CG: *C. glycolicum*; CDi: *C. difficile*.

## Genome properties

**Figure 6:**
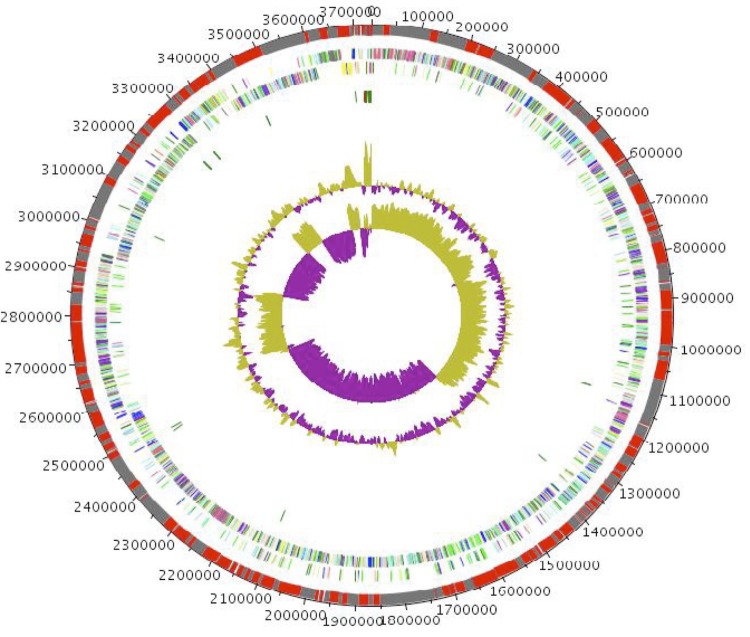
Graphical circular map of the chromosome. From the outside in, the outer two circles show open reading frames oriented in the forward and reverse directions (colored by COG categories), respectively. The third circle marks the rRNA gene operon (red) and tRNA genes (green). The fourth circle shows the G+C% content plot. The inner-most circle shows the GC skew, purple and olive indicating negative and positive values, respectively.

The genome of *C. dakarense* sp. nov. strain FF1^T^ is 3,735,762 bp long (1 chromosome, but no plasmid) with a 27,98% G + C content of (Figure 6 and Table 4). Of the 3,916 predicted genes, 3,843 protein-coding genes, and 73 were RNAs. Eight rRNA genes (one 16S rRNA, one 23S rRNA and six 5S rRNA) and 65 predicted tRNA genes were identified in the genome. A total of 2,769 genes (72.05%) were assigned a putative function (by COG or NR blast). Two hundred ninety-eight genes were identified as ORFans (7.75%). The remaining 515 genes were annotated as hypothetical proteins (13, 40%). The distribution of genes into COGs functional categories is presented in Table 4. The properties and the statistics of the genome are summarized in Tables 4 and 5.

**Table 4:** Nucleotide content and gene count levels of the genome.

**Attribute**	**Value**	**% of total^a^**
Genome size (bp)	3,735,762	100
DNA coding region (bp)	3,239,020	86.70
DNA G+C content (bp)	1,045,424	27.98
Total genes	3,916	100
RNA genes	73	1.86
Protein-coding genes	3,843	98.14
Genes with function prediction	2,769	72.05
Genes assigned to COGs	2,849	74.13
Genes with peptide signals	410	10.67
Genes with transmembrane helices	1,016	26.44

^a^ The total is based on either the size of the genome in base pairs or the total number of protein coding genes in the annotated genome

**Table 5:** Number of genes associated with the 25 general COG functional categories.

**Code**	**Value**	**%age**^a^	**Description**
J	171	4.45	Translation
A	0	0	RNA processing and modification
K	325	8.46	Transcription
L	158	4.11	Replication, recombination and repair
B	1	0.03	Chromatin structure and dynamics
D	34	0.88	Cell cycle control, mitosis and meiosis
Y	0	0	Nuclear structure
V	111	2.89	Defense mechanisms
T	225	5.85	Signal transduction mechanisms
M	165	4.29	Cell wall/membrane biogenesis
N	58	1.51	Cell motility
Z	0	0	Cytoskeleton
W	0	0	Extracellular structures
U	45	1.17	Intracellular trafficking and secretion
O	95	2.47	Posttranslational modification, protein turnover, chaperones
C	194	5.04	Energy production and conversion
G	248	6.45	Carbohydrate transport and metabolism
E	248	6.45	Amino acid transport and metabolism
F	88	2.29	Nucleotide transport and metabolism
H	117	3.04	Coenzyme transport and metabolism
I	72	1.87	Lipid transport and metabolism
P	181	4.71	Inorganic ion transport and metabolism
Q	52	1.35	Secondary metabolites biosynthesis, transport and catabolism
R	386	10.04	General function prediction only
S	261	6.79	Function unknown
-	994	25.87	Not in COGs

^a^ The total is based on the total number of protein coding genes in the annotated genome.

## Comparison with the genomes from other *Clostridium* species

The genome sequence of *Clostridium sp.* is currently available for more than seventy-five *Clostridium* species. Here we compared the genome sequence of *C. dakarense* strain FF1^T^ with than those of *C. bartlettii*, *C. beijerinckii*, *C. cellulovorans*, *C. difficile*, *C. glycolicum*, *C. perfringens*, *C. saccharolyticum*, *C. senegalense*, and *C. thermocellum.*

The draft genome sequence of *C. dakarense* strain FF1^T^ is smaller than those of *C. cellulovorans*, *C. beijerinckii*, *C. senegalense*, *C. saccharolyticum*, *C. thermocellum*, *C. difficile, C. glycolicum* (3.73, 5.26, 6.0, 3.89, 4.66, 3.84, 4.3 and 3.99 Mb, respectively) but larger than those of *C. perfringens* and *C. bartletii* (3.26 and 2.97 Mb, respectively). The G+C content of *C. dakarense* is lower than those of *C. cellulovorans*, *C. beijerinckii*, *C. perfringens*, *C. saccharolyticum*, *C. thermocellum*, *C. difficile* (31.2, 29.9, 28.4, 45, 39 and 29.1%, respectively) but higher than those of *C. bartlettii, C. glycolicum* and *C. senegalense* (28.8, 28 and 26.8%, respectively). The gene content of *C. dakarense* is larger than those of *C. thermocellum*, *C. senegalense, C. perfringens*, *C. glycolicum*, *C. bartlettii* (3,916, 3,173, 3,761, 2,876, 3,840 and 2,787, respectively) and smaller than those of *C. cellulovorans*, *C. beijerinckii*, *C. saccharolyticum* and *C. difficile*, (4,501, 5,243, 4,154 and 4,019, respectively). The ratio of genes per Mb of *C. dakarense* is larger to those of *C. cellulovorans*, *C. beijerinckii*, *C. senegalense*, *C. saccharolyticum*, *C. thermocellum*, *C. difficile*, *C. bartlettii*, *C. glycolicum* and *C. perfringens* (1,049, 856, 874, 966, 891, 826, 934, 938, 962 and 882, respectively).

The number of orthologous genes shared between *C. dakarense* and other compared *Clostridium* species has been summarized in Table 6. The average percentage of nucleotide sequence identity ranged from 62.05 to 74.5% among previously published *Clostridium* species, and from 61.94 to 75.7% between *C. dakarense* and other studied *Clostridium* species, thus confirming its new species status.

## Conclusion

On the basis of phenotypic, phylogenetic and genomic analyses, we formally propose the creation of *Clostridium dakarense* sp. nov. which contains strain FF1^T^. This bacterium strain has been isolated from the fecal flora of a 4-months-old Senegalese child suffering from gastroenteritis.

### Description of *Clostridium senegalense* sp. nov.

*Clostridium dakarense* (da.kar.e′n.se. L. gen. neutr. n. *dakarense*, pertaining to, or originating from Dakar, the capital of Senegal, where the type strain was isolated).

Colonies were 1.5 mm in diameter on blood-enriched Columbia agar and Chocolate agar + PolyViteX. Cells are rod-shaped with a mean diameter of 1.2 μm. Optimal growth is achieved anaerobically. No growth is observed in aerobic conditions. Growth occurs between 25-37°C, with optimal growth observed at 37°C, in medium 5% sheep blood-enriched Columbia agar. Cells stain Gram-positive, are endospore-forming, and motile. Catalase, oxidase, urease, indole and nitrate reduction activity are absent. Arginine dihydrolase, N-acetyl-β-glucosanimidase and pyroglutamic acid arylamidase activity are present. Cells are susceptible to amoxicillin, metronidazole, vancomycin, imipenem and rifampicin but resistant to trimethoprim/sulfamethoxazole.

The G+C content of the genome is 27.98%. The 16S rRNA gene sequence and whole-genome shotgun sequence of *C. dakarense* strain FF1^T^ (= CSUR P243 = DSM 27086) are deposited in GenBank under accession numbers KC517358 and CBTZ00000000, respectively. The type strain FF1^T^ (= CSUR P243 = DSM 27086) was isolated from the fecal flora of a 4-months-old child in Dakar, Senegal.
